# Reduced Slit Rolling Power in Rebar Steel Production

**DOI:** 10.3390/ma16052104

**Published:** 2023-03-05

**Authors:** Rashid Khan, Sabbah Ataya, Islam Elgammal, Khamis Essa

**Affiliations:** 1 Department of Mechanical & Industrial Engineering, College of Engineering, Imam Mohammad Ibn Saud Islamic University, Riyadh 11432, Saudi Arabia; 2 Al Ezz Flat Steel, Soukhna, Suez, Egypt; 3 School of Mechanical Engineering, University of Birmingham, Edgbaston, Birmingham B15 2TT, UK

**Keywords:** rebar steel, rolling, slitting, power reduction, finite element simulation

## Abstract

The rolling process of rebar steel production is one of the well established manufacturing processes; however, it should be subjected to revision and redesign for productivity enhancement and power reduction throughout the slit rolling process. In this work, slitting passes are extensively reviewed and modified for the attainment of better rolling stability and reduction in power consumption. The study has been applied for grade B400B-R Egyptian rebar steel, which is equivalent to steel grade ASTM A615M, Grade 40. Traditionally, the rolled strip in the rolling pass is edged before implementing a slitting pass using grooved rolls; this produces a single barreled strip. This single barrel form causes instability in the next slitting stand on the pressing by the slitting roll knife. Multiple industrial trials are attempted to achieve the deformation of the edging stand using a grooveless roll. As a result, a double barreled slab is produced. In parallel, finite element simulations of the edging pass are performed using grooved and grooveless rolls, and similar slab geometry with single and double barreled form are produced. In addition, further finite element simulations of the slitting stand are execute using idealized single barreled strips. The power calculated by the FE simulations of the single barreled strip is (245 kW), which is in acceptable agreement with the experimental observations in the industrial process (216 kW). This result validates the FE modeling parameters such as material model and boundary conditions. The FE modeling is extended to the slit rolling stand of a double barreled strip, which was previously produced by the grooveless edging rolls. It is found that the power consumption is (165 kW) 12% lower than the power consumed (185 kW) for slitting the single barreled strip.

## 1. Introduction

Rolling is the most common bulk deformation processes, and rebar steel production represents the largest part of long rolled products. The high demand of ribbed steel is mainly due to its application in reinforced concrete structures. Rebar steel is usually produced in hot rolling mills using grooved rolls [1]. In general, the process of grooved hot rolling of steels involves the following [2]: (1) preheating steel billet to a temperature of about 1200 °C, and (2) rolling the billet by pressing it repeatedly in between the pairs of grooved rolls that have a controlled gap, as per the roll pass design.

**Figure 1:**
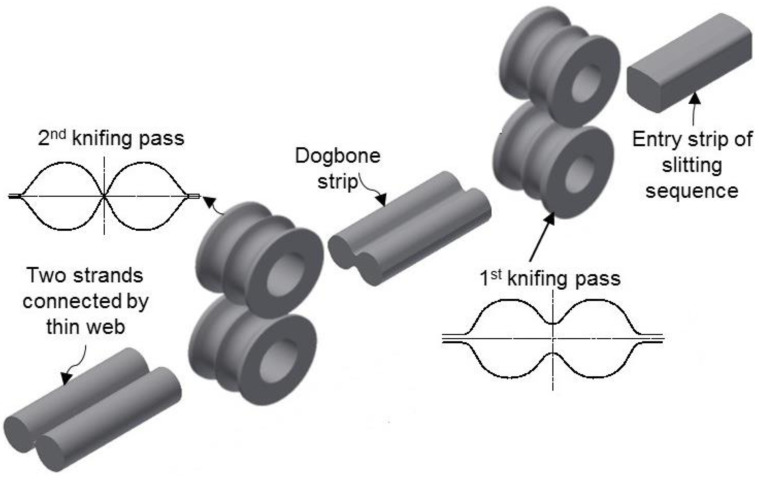
Slitting passes of the single barreled strip first and second knifing type passes in the slit rolling subsequence; commonly named ‘dogbone’ pass and ‘slitting’ pass, respectively.

Along with other material and design related issues, cost reduction in rolling long products is equally imperative. Many researchers, since five decades ago, have extensively investigated this critical matter [3]. The foremost outcome of the intensive research originated through slit rolling. The slit rolling process is one of the processes aiming to increase productivity while reducing production cost. It is referred to a rolling subsequence in which an incoming strip is longitudinally severed into two strands [4]. For a multi slit rolling process [5], the strip is severed into three, four, or five strands. In both cases, the severed strands are rolled in parallel passes, downstream to the slitting subsequence. Recent slit rolling subsequences are based on two knifing type passes [6], gradually slitting the incoming strip into semi-connected strands, as schematically shown in Figure 1. A complete severance is achieved with the aid of the ‘diverging displacement’ of the semi-connected strands. This is induced by a specially designed roll pass, profile, or slitter roller guide, located at the end of the slitting subsequence. Although slit rolling has proved to be an efficient and effective process, there is still a need to review its economical aspect.

**Figure 2:**
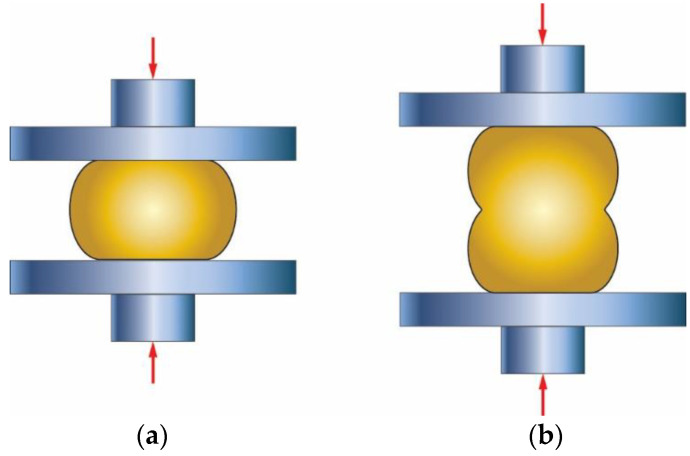
Deformation modes in compression; (**a**) single barrel, **(b**) double barrel, adopted from [7].

The deformation behavior of the material varies with the geometry of the slap once subjected to compression. Useful information about deformation and failure modes can be extracted from compression tests, as shown in Figure 2. Friction is a source of inconsistent deformation in compression of ductile materials and represents a resistance to lateral flow at the mating surfaces, which leads to barreling or bulging of the cylindrical surface. It is reported [7,8] that if the cylindrical specimens have length-to-diameter ratios (L/D) less than 2.0, a single barrel forms, as shown in Figure 2a. Barreling indicates that the deformation is not uniform, and thus the stress and strain vary throughout the test specimen. At the same friction circumstances with an L/D of order 2.0, a double barrel forms, Figure 2b.

Due to the high cost of experimentation and industrial trials, studies of bulk deformation processes are associated with extensive application of the finite element simulation. To carry out such FE simulation with acceptable and reliable results, applicable material laws are highly needed [3,9]. There are many proposed material laws presenting the materials’ behavior at different temperatures and strain rates [10,11,12,13]. However, it is difficult to find models for a specific material. Therefore, several finite element analyses adopt user defined material models (UMAT) based on their specific requirements. In this, model parameters are usually extracted from experimental testing like uniaxial tensile and compression using standard procedures. However, this procedure is computationally and technically costly. Alternatively, published experimental data of the nearest material can also be utilized for defining the material flow rule in the finite element procedure. This approach is not cumbersome, however, it may contain a significant magnitude of error, which probably needs to be addressed in the results’ analysis.

Many researchers studied the slitting process from different point of views. Sung et al. [14] studied the effect of the two roll grooves on the final rebar geometry, presenting rebar sizes which differ from the ASTM sizes. In a rolling flat small section, Lundberg [4] has presented the idea of using an edging flat roll instead of a grooved roll to avoid some troubles associated with grooved rolls such the excess of material removal on dressing after wear of grooved rolls. The design of the slitting pass for two, three, and four bars has been studied by Turczyn et al. [15]. In the case of two bars, the cross section of the entry strip to the slitting pass was a rhombus shape. This rhombus strip is even more challenging for the stability of the roll knife. Lambiase [16] numerically studied the possible shapes resulting from the slitting pass using an entry bastard oval (quasi rectangular with a flat top) strip.

The prime aim of the current work is to numerically investigate the possibilities of power reduction by replacing the grooved rolls (in the edging rolling stand of rebar rolling) with a grooveless in slit rolling process. Initially, a strip edging—using a grooveless rolls—is examined to prove it is better than the single barrel form of strip obtained through grooved edging rolls. Then, the numerical models of single and double barrels are developed using finite element software ABAQUS using almost identical geometrical, material, and physical boundary conditions as in industrial practice. Afterwards, the numerical results of the single barrel strip are compared and verified with the experimental observations. Finally, double barrel simulations are executed using verified numerical parameters from the single barrel form.

## 2. Material and Method

**Figure 3:**
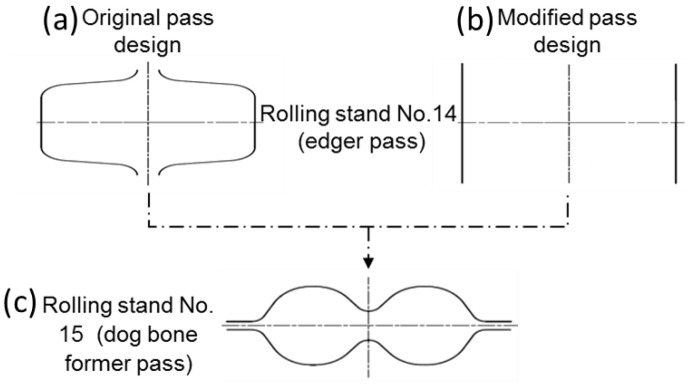
Schematic of the roll pass of stand 14. (**a**) Original grooved rolls, (**b**) modified grooveless roll, and (**c**) stand 15 with dogbone pass.

To study the significance of a roll pass design modification at stand 14 by changing the rolls from grooved into grooveless (as shown in Figure 3a,b), samples were taken out from the rolled strip after stand 14 and before entering the first slitting former pass at stand 15 which have dog bone shape (Figure 3c). The samples (15 mm long) were cut off, mounted in an epoxy mold, and grinded using SiC emery papers till grade 1000.

**Figure 4:**
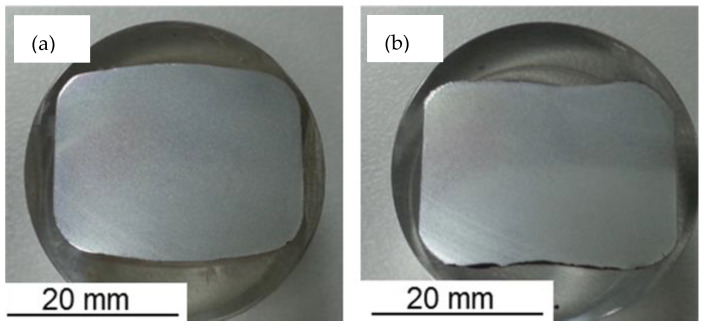
Images of real strips as obtained after rolling through stand 14. (**a**) Strip deformed by grooved rolls, (**b**) strip deformed by grooveless rolls.

Figure 4 shows the strips taken out from the rolling bar at stand 14. After this, the slitting stand produces two strands of rebar steel with a nominal diameter of 12 mm. In Figure 4a, the strip has a central bulged profile, where the peak thickness at the middle zone is measured as 19 mm and decreases laterally in between 16 mm and 17 mm at the curved fillet. The inclination angle with the transversal axis of the grooved edger pass increases the central bulging. In Figure 4b, the lateral concentered mass is formed at the expense of the middle zone bulging, where the height at the lateral and middle zones is 18.7 mm and 17.5 mm, respectively. The entry strip to the stand 14 has the dimensions of 16.2 mm height and 32.9 mm width, and the temperature is 1100 °C.

Different steel grades are manufactured using current rolling mills for producing multiple products with different final dimensions, especially rebar with final nominal diameters of 12 and 16 mm. This study is conducted on one of the frequently produced steel grades with chemical compositions as listed in Table 1. It is rolled Egyptian steel Grade B400B-R according to the Egyptian Standard ES 262-2/2015, which is equivalent to ASTM 615M Grade 40 [17].

In different studies, it is found that Misaka’s model is one of the preferred material models at the current deformation conditions [18]. In this study, the plastic parameters are determined from the Misaka’s equation, Equation (1), with strain and strain rate dependent flow stress, according to the model reported by Byon et al. [18], that is stated below:

(1) $$\sigma =9.8\exp\left(0.126-1.75C+0.594C^{2}+\frac{2851+2968C-1120C^{2}}{T}\right).\;\varepsilon {\;}^{0.21}\varepsilon^{}{}^{●0.13}$$

where, *σ* is the flow stress (MPa), *T* is the temperature (°C), ε is the strain (--), $\varepsilon^{●}$ is strain rate (s^−1^), and *C* is the carbon content (wt.%).

**Figure 5:**
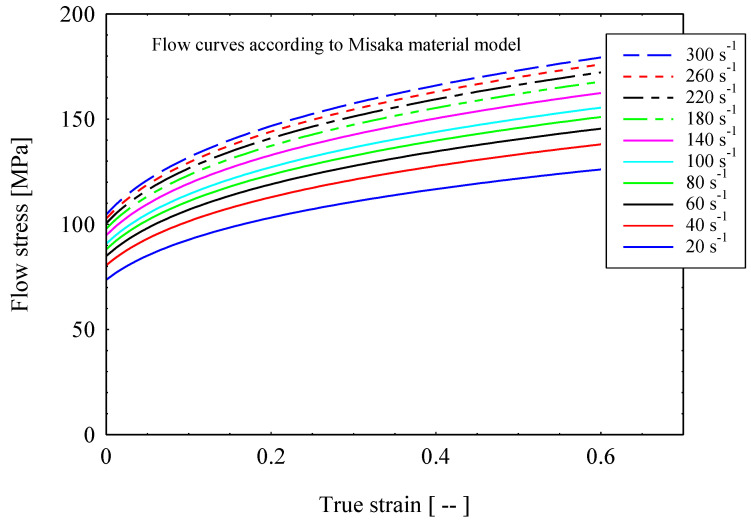
Flow curves according to Misaka’s material model as a function of strain and strain rate (20–300 s^−1^) at a temperature of 1100 °C.

The advantage of this model is the consideration of carbon content as a major affecting alloying element besides the processing conditions, i.e., temperature, strain, and strain rates. The elastic modulus is considered as temperature dependent [19], which is around 95 GPa at 1100 °C, and the Poisson ratio is 0.35. The contact interaction among the rollers and billet is modeled by a penalty contact with a tangential friction coefficient equal to 0.3, as an output of the presented friction equation by Lambiase [16]. The material behavior for the FE analysis is defined based on the elastic–plastic deformation model of one of the rebar steel grades, as shown in Table 1. Figure 5 shows the flow curves according to Misaka’s material model as a function of strain and strain rate (20–300 s^−1^) at a temperature of 1100 °C.

Byon et al. [18] reported that the strain rates at the last four finishing stands of the block mill are in the range of 100−400 s^−1^ at a temperature range from 900 °C to 1050 °C. In hot rolling with strain rate range up to 100 s^−1^, the deformation process can be considered as isothermal, as stated by El-Magd et al. [12], and the temperature effects can be ignored. However, in the current hot rolling processes, the average strain rate reached 200 s^−1^, which still could be considered as the moderate strain rate and isothermal conditions that can be applied for simplification. The transition from isothermal to adiabatic deformation depends on the strain (as a source of temperature rise), strain rate, and the material thermal properties, as reported by El-Magd [13]. Therefore, the effect of extreme conditions of both strain and strain rate under the roll knife apex arises. Thus, ductile fracture parameters are going to be measured and compared during the strand severance step.

## 3. Finite Element Modeling

**Figure 6:**
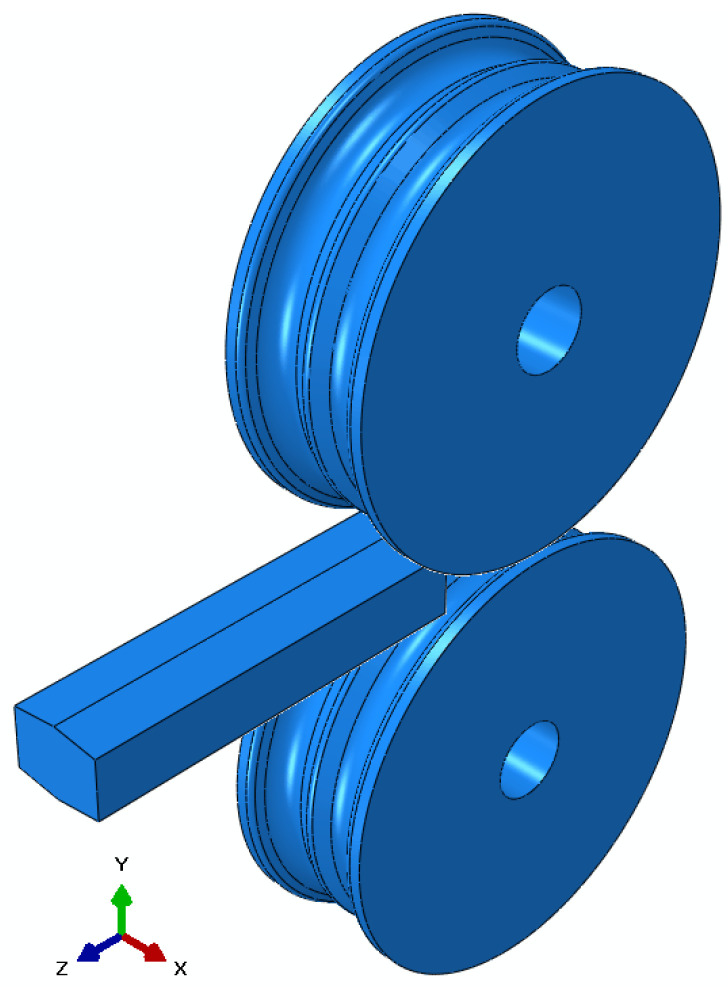
Finite element model of first slit rolling stage.

**Figure 7:**
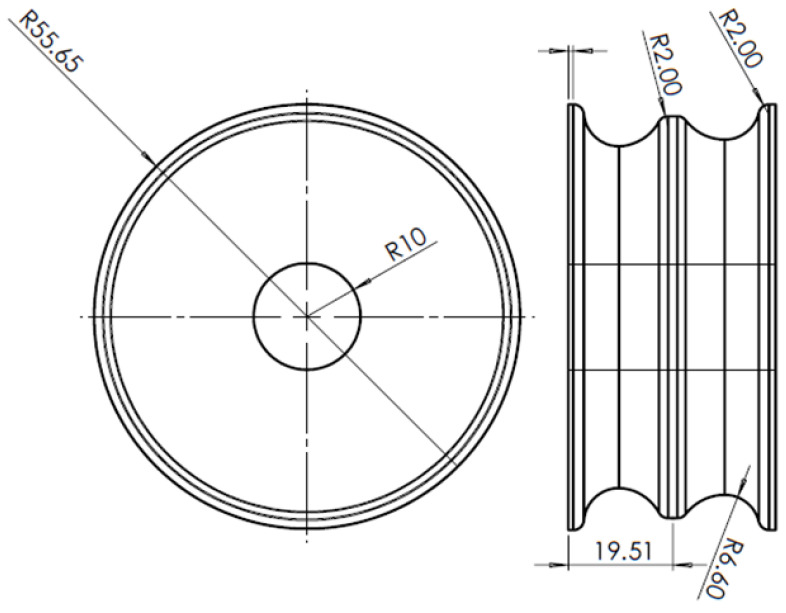
Roller front and side views with geometric dimensions. All dimensions are in mm.

**Figure 8:**
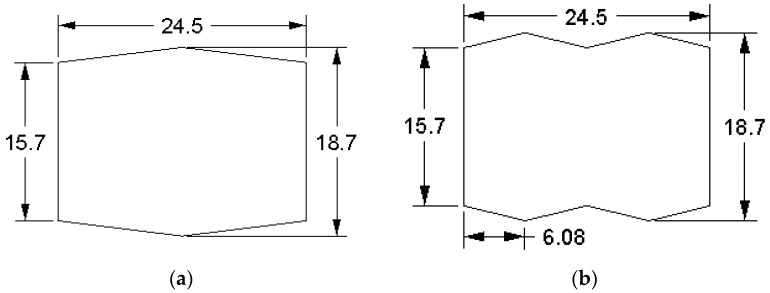
Two billet designs: (**a**) single barrel, (**b**) double barrel (dog bone).

**Figure 9:**
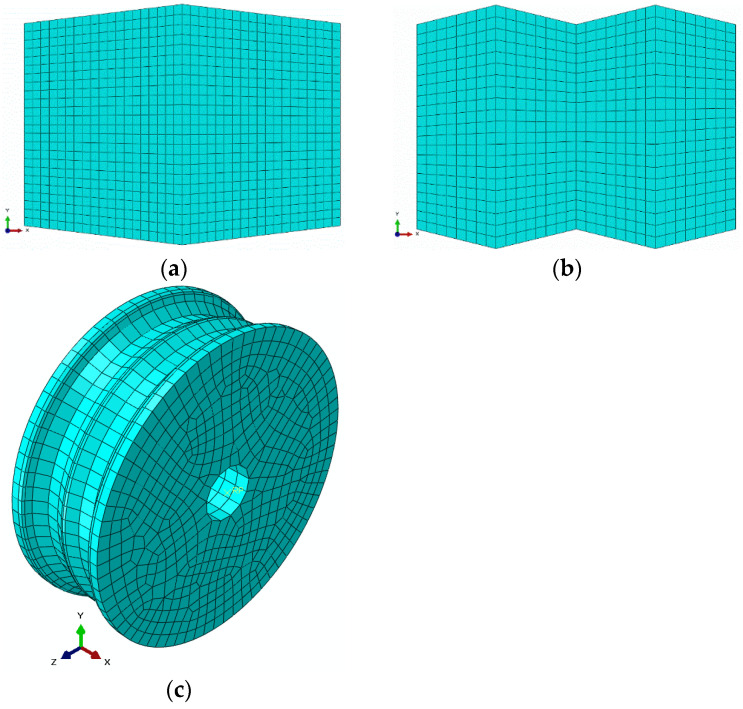
Discretized models of (**a**) single barrel, (**b**) double barrel, (**c**) roller.

The finite element method is adopted for predicting the effects of billet shape on the overall power requirement in the slit rolling process. The finite element models are developed using the commercial finite element package ABAQUS. The finite element model of the first slit rolling stage is shown in Figure 6. A detailed view of the roller and dimensions are mentioned in Figure 7. The two billet models, A and B, are adopted in the current study, as shown in Figure 8. The prime difference between the two billet designs is in the geometry of the entry strip, which undergoes the rolling of a similar profile through the dogbone pass (roller). To minimize the effect of idealization of the billets as fillet free corners, the area is kept equal to the real strips shown in Figure 4. In order to mimic the experiments and deformation verification, the assembly is considered as whole, as shown in Figure 6. However, in future work, a twofold planar symmetry will be implemented. The billet models are idealized through “fillet free” corners, so mapped or structured mesh can be generated without geometry partitioning. An elastic plastic with a nonlinear strain hardening material model is defined for the grade B400B-R Egyptian steel at 1100 °C as billet material. The chemical composition of this steel is defined earlier. The Misaka’s equation’s, Equation (1), is used to define the material flow curves at a strain rate range of 20–300 s^−1^ and temperature of 1100 °C, as shown in Figure 5. The other material parameters at 1100 °C are taken as follows: Young’s modulus = 95 GPa, Poisson ratio = 0.35, and density = 7800 kg/m^3^. The surface-to-surface contact interaction between rollers and billet is modeled through a penalty contact with a tangential friction coefficient of 0.35 [16]. In boundary conditions, the billet longitudinal velocity along the z-axis is set at 150 mm/s, which is equivalent to 47.1 radians/s angular velocity of rollers. All other degrees of freedom are fixed for both rollers and billet. The rollers are modeled as discrete rigid bodies using 4node 3D bilinear rigid quadrilateral (R3D4) elements. While the billet is modeled as a deformable body using an 8-node linear brick hourglass control element with reduced integration (C3D8R). The number of elements in billets A and B are 140,448 and 84,000 with the average aspect ratios of 1.29 and 1.09, respectively. In roller, the number of elements is 2264 with the average aspect ratio of 1.29. The finite element models are shown in Figure 6 and Figure 9. A fully dynamic implicit integration scheme (simulation algorithm) is used; the maximum number of increments are 10,000 and the initial increment size is 0.001, without mass scaling.

## 4. Results and Discussion

### 4.1. Strip Edging for Preslitting

**Figure 10:**
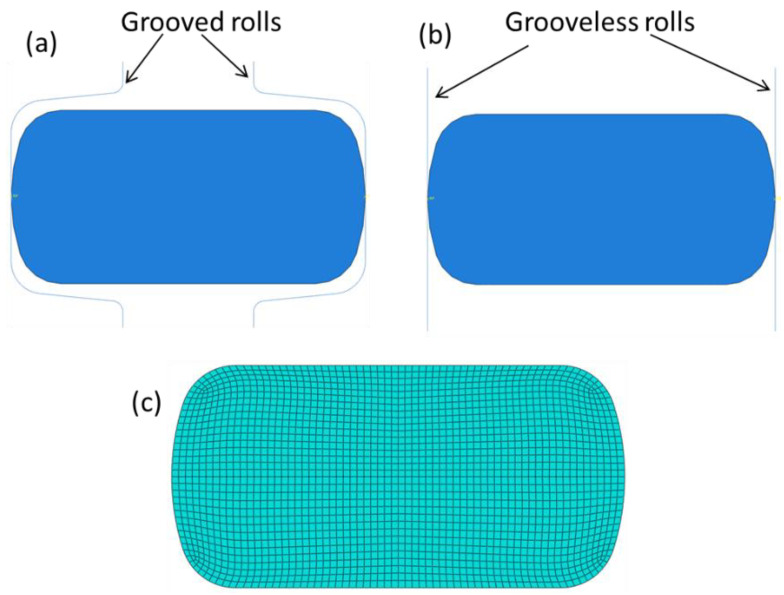
Idealization of the entry strip to stand 14. (**a**) Strip in the grooved roll, (**b**) strip in the grooveless roll, and (**c**) meshed strip for FE simulation.

Examination of formation either single or double barrel form by the FE method is conducted using a 2D model, as shown in Figure 10. The model geometry is equivalent to the strip, resulting from stand 13 and entering the edging pass, having dimensions of 16.2 mm height (H) and 32.9 mm width (W) with an aspect ratio (W/H) of 2.03. The model is meshed into 2266 elements (Figure 10c) of reduced quadratic plane strain type. Figure 10a shows the idealized sample entering stand 14 to be edged using grooved rolls. Alternatively, Figure 10b shows similar samples prior to deformation using grooveless rolls, having alike dimensions as prescribed above. The strip has been numerically simulated by through deformation and reduction in width (W) from 32.9 to 24.5 mm.

**Figure 11:**
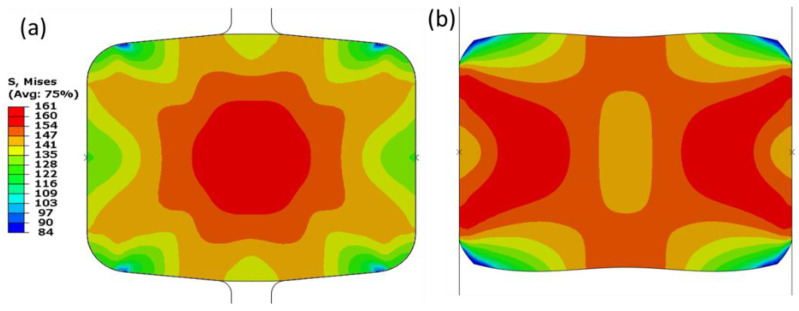
Von Mises stress distribution on the deformed strip after strip deformation by stand 14. (**a**) Strip deformed by grooved rolls; (**b**) strip deformed by grooveless rolls.

Figure 11 shows the stress distribution of the deformed strip under the edging using grooved and grooveless rolls. The deformation restriction caused by the grooved roll has forced the material to flow from the lateral sides to the strip center (central bulging) of the sample, as presented in Figure 11a. The stress distribution shows a concentrated pattern of high stress in the middle of the sample, which changes grooveless rolls into shear bands or double rounded top isosceles triangles, as shown in Figure 11b. This represents a typical deformation pattern of material once subjected to compression loads, especially under quasi static deformation condition [20].

**Figure 12:**
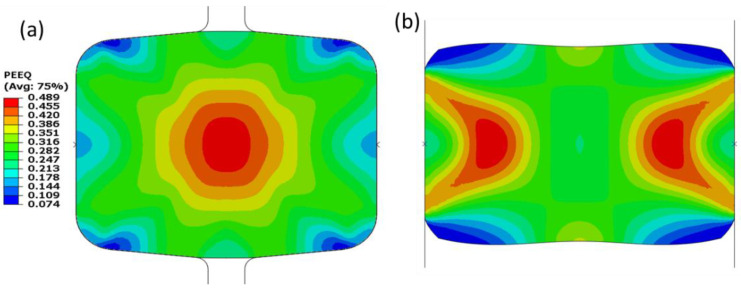
Equivalent plastic strain distribution on the deformed strip after strip deformation by stand 14. (**a**) Strip deformed by grooved rolls, (**b**) strip deformed by grooveless rolls.

The strain distribution (Figure 12) indicates a similar pattern as stress (Figure 11). Moreover, deformation using a grooveless roll (Figure 12b) showed larger dead or slightly deformed regions at the top and bottom areas. This is mainly due to the unrestricted lateral flow (to the top and bottom of the strip) of the deformed material, forming a dogbone shape.

### 4.2. Finite Element Simulations of Preslitting Stand

**Figure 13:**
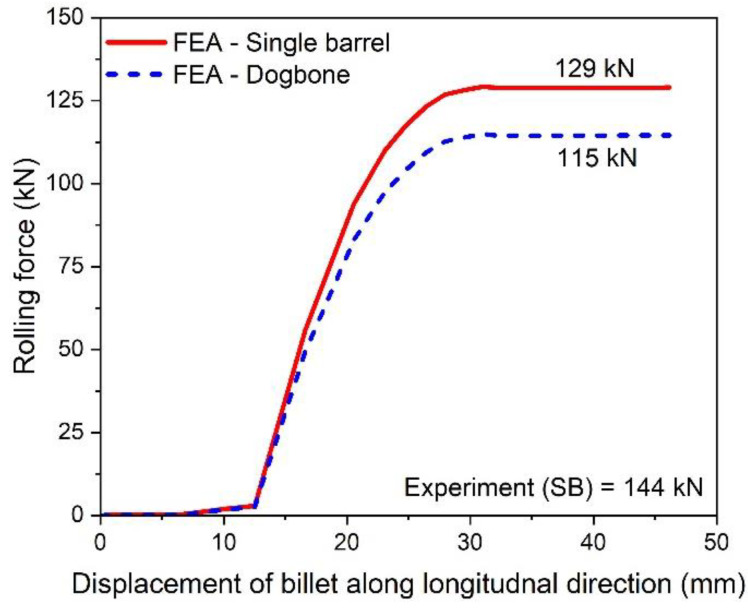
Variation of rolling force with billet displacement for a single and double barrel.

**Figure 14:**
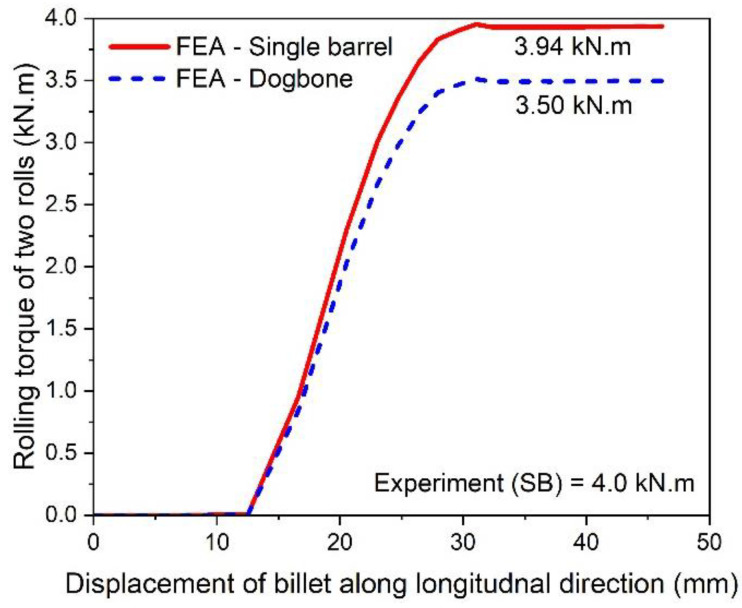
Variation of rolling torque with billet displacement for single and double barrel.

**Figure 15:**
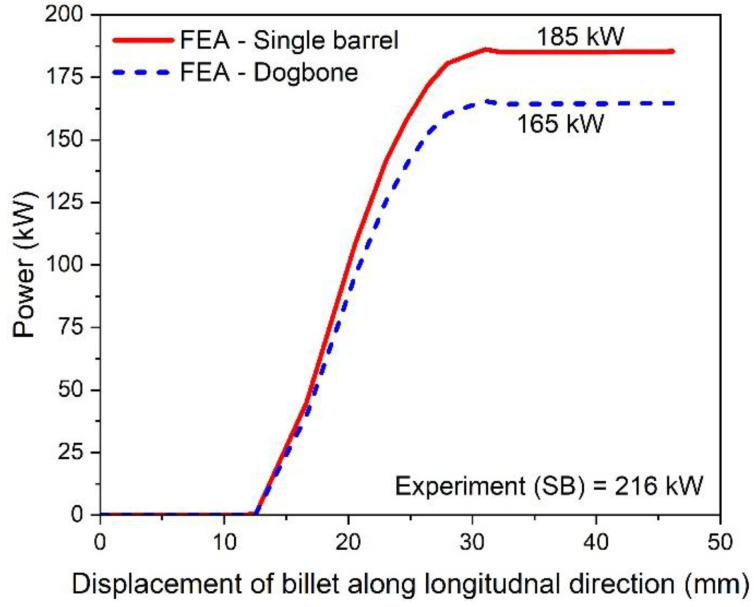
Power requirement for single and double barrels.

**Figure 16:**
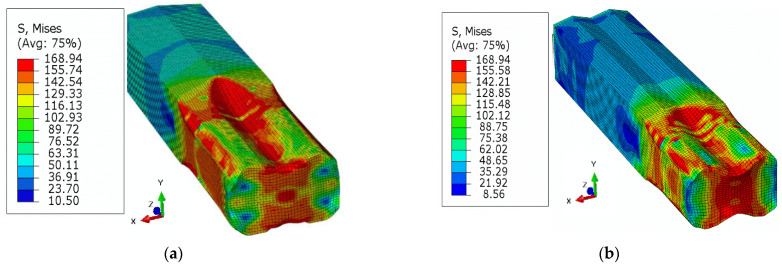
Von Mises stress distribution: (**a**) single barrel, (**b**) double barrel.

**Figure 17:**
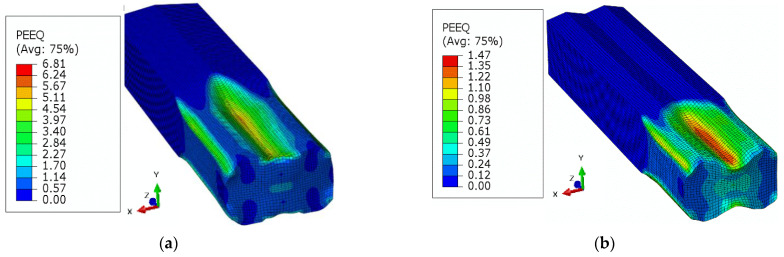
Equivalent plastic strain (PEEQ) distribution: (**a**) single barrel, (**b**) double barrel.

**Figure 18:**
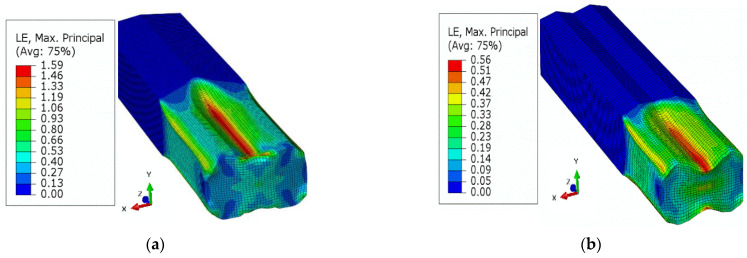
Maximum principal logarithmic strain distribution: (**a**) single barrel, (**b**) double barrel.

**Figure 19:**
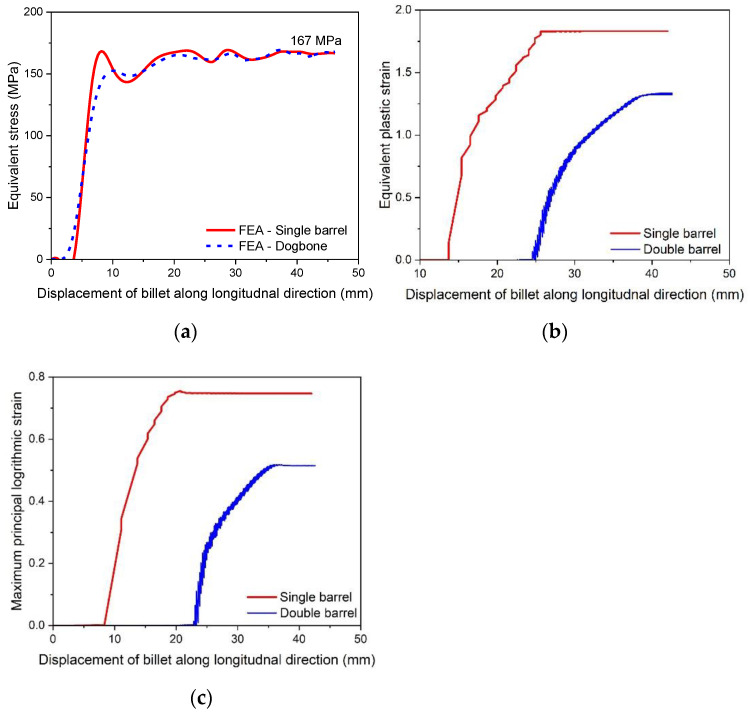
Material deformation parameters for single and double barrels, (**a**) von Mises stress, (**b**) equivalent plastic strain, (**c**) maximum principal logarithmic strain.

Finite element simulations are executed for single and double barrel (dog bone) assembly for investigating the rolling force, torque requirement, power consumption, equivalent stress, contact pressure, and equivalent plastic strain, as represented by Figure 13, Figure 14, Figure 15, Figure 16, Figure 17, Figure 18 and Figure 19. The rolling force, F of both types of barrels can be estimated by Equation (2) as follows:

(2) $$F=\sigma_{f}wL\;,$$

where $\sigma_{f}$ is the flow stress, and w and L are, respectively, the width and contact length of the barrel. The flow stress can be estimated through Equation (3) [21] as follows:

(3) $$\sigma_{f}=\frac{K\epsilon^{n}}{1+n}.$$

Here, K and n are material parameters, which can be extracted from the stress strain curve of material by using linear regression (linear curve fitting) of the data points between $ln(\sigma_{f})$ and $ln\left(\epsilon \right)$. The values of parameters are found as K=195.04 MPa, and n=0.2157. The contact length, Equation (2), is calculated by Equation (4) [21] as follows:

(4) $$L=\sqrt{R\left(t_{i}-t_{f}\right)}\;,$$

where, R is the radius of the roller (55.6495 mm), $t_{i}$ is the initial, while $t_{f}$ is the final thickness of the barrel. The value of $t_{i}$ is 18.7 mm, while the initial width of barrel, w is taken as 24.5 mm. The finite element results of the variation of rolling force with respect to the displacement of the billet for single and double barrels are shown in Figure 13. In addition, experimental observations of the single barrel (SB) are compared with finite element analysis (FEA) results of the SB and dogbone or double barrel (DB) samples.

The simulation results of the rolling force of a single barrel predicted experimental observations well, with a difference of 11.6%. The possible reasons for this variation may include the following: (i) approximation of fillet free billet geometry, (ii) assumption of isotropic and homogenous material in FEA, and (iii) consideration of linear friction model in FEA, while nonlinear friction behavior exists in reality. Moreover, the rolling force in case of a double barrel is lower (12.17%) than for a single barrel, which shows promising benefits of using a double barrel in stage 15 of the slit rolling process.

The required torque (N.m) of the rollers can be estimated through Equation (5) as follows:

(5) $$T=F*\left(\frac{L}{2}\right)\;$$

The variation in the rolling torque of two rollers is shown in Figure 14. It is clear that the rolling torque in a double barrel setup is lower than in case of the single barrel. The difference is approximately 12.6%, which also shows prominent reduction in the torque requirement for a double barrel billet shape. Furthermore, the simulation results of the single barrel setup are close enough to the experimental findings (error is ~1.52%). After calculating the torque, the power can be estimated through Equation (6) [21] as follows:

(6) P=2πNFL , 

where, power is in watts, and N is the rotational speed of the roll, which is taken as 47.1 rad/s or 449.77 RPM, as used in the slit rolling process. The power requirements of single and double barrels are illustrated in Figure 15. It is found that the required power for a double barrel is significantly reduced compared to for a single barrel (~12.12%). This could be considered as meaningful in a large industrial slit rolling setup where a unit power consumption makes huge difference in the overall cost of the product. The mechanical advantage—reduction in the rolling force and power of a double barrel steup could be mainly due to its geometry. As mentioned in Figure 8a,b, the initial rolling thickness of single and double barrels are 18.7 and 15.7 mm, respectively. This reduction favors double barrels in terms of overall power requirement to achieve the final thickness. In addition, the contact region in between the single barrel and roller is a crest, while it is a trough in a double barrel setup. This profile variation may also reduce its power requirements. This is one of the main outcomes of the current research, in which it is proven that the proposed geometry (double barrel) of the billet could increase the efficiency of the process. These results can be further investigated by improving the finite element model or the geometrical features of double barrel setups; this would be included in future research. In addition, as referred from Figure 15, the finite element results predict the experimental value of the power well. However, the error is 16.75%, which is considered high. Similar reasons, as mentioned above in case of rolling force, could lead to this variation.

In addition to the process parameters, the material related factors are of the similar importance. The equivalent stress, Equivalent Plastic Strain (PEEQ), and maximum principal logarithmic strain (MPLS) are categorized as crucial parameters for the slit rolling process. These parameters’ contour plots for single and double barrels are shown in Figure 16, Figure 17 and Figure 18. Moreover, the variation of von Mises stress, PEEQ, and MPLS, with respect to billet displacement, are graphically shown in Figure 19. It is noted that these parameters represent the values of a single node, which is assumed to have maximum values of stress and strain since it lies at the direct contact region between billet and roller. It is evident that the maximum von Mises stress in single and double barrels has the similar magnitude of 168 MPa. However, there is a significant difference in the values of PEEQ and MPLS. The PEEQ for single and double barrels is 1.82 and 1.33, respectively, while the MPLS is, respectively, equal to 0.75 and 0.51.

### 4.3. Deformation Behaviour at the Preslitting Stand

**Figure 20:**
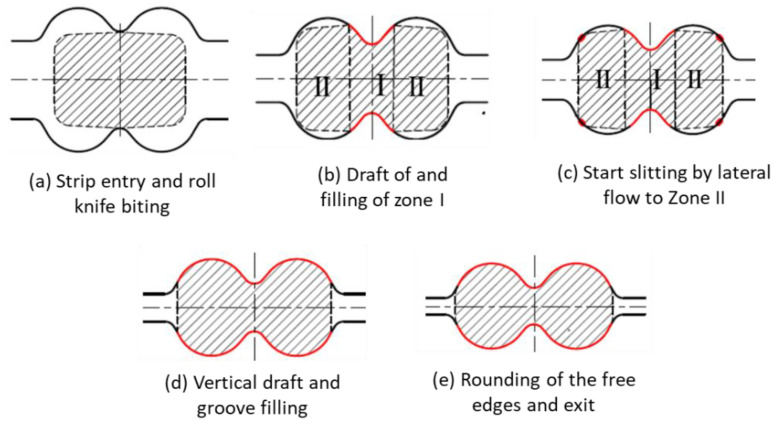
Schematic representation of the deformation stages at stand 15 (preparation stage for slitting) of the single barreled strip resulting from the edging process at stand 14.

The progressive deformation stages under the dog bone pass (stand 15) are presented in Figure 20 in order to show the variation in the deformation steps of the preslitting of single and double barrel strips. The deformation at this stand starts with cutting the entering strip through the apex roll, Figure 20a. Afterwards, the strip is pressed by the roll knife to spread it laterally in order to fill zone I, as shown in Figure 20b. Upon continual pressing of the strip, the extreme sides turn out to be in direct contact with the internal sides of the groove. On deeper slitting, the knifing action causes an indirect side draft for zones II, as illustrated in Figure 20c. Then, while rolling the vertical draft, the strands’ zones (I, II) are combined to the overall draft, and zones II are completely occupied (Figure 20d). Finally, the deformation at stand 15 is completed at the exit plane of the rolling pass (Figure 20e) while rounding the free edges of the strip sides.

In the first stage of deformation (Figure 20a,b), the drafted zone (zone I) under the knife apex is deformed (mostly into spread) at the expense of an effective elongation. This occurs due to the coherence of the strip parts, which tends to equalize the speed of different parts of the strip that are exposed to a differential draft. Hence, the pulling down of less drafted parts (extreme zones) to the heavily drafted part (central zone) is stabilized by a forced lateral spread, i.e., the mass flows from the latter zone to the former one. The consumed work, during such a forced spread, is considered ineffective because it increases the stress localization and intensification at the knife apex (Figure 16a,b). Sever biting action (Figure 20a) is required for the deformation of a single barreled strip, produced by grooved edging rolls (Figure 4a). Upon deformation of the double barreled strip, generated by grooveless rolls (Figure 4b), biting would become much easier as its slitting pass begins with the second or the third step, shown in Figure 20b,c. The initial geometrical coincidence of the strip pass contacting surfaces (Figure 20b) increases effective elongation while decreasing forced spread by accelerating the full contact of the strip parts to the roll pass [22]. Figure 20 can also explain why the deformation of a single barreled strip is associated with higher levels of stress strains than that of double barreled strips. Suppressing the increased height (H) in the middle of the single barreled strip requires implementing more strain just to make it resemble that of the double barreled strip.

In addition to reducing the deformation steps at the first slitting pass, introducing a double bulged strip is very beneficial in reducing the sever wear of the rolls [23]. More specifically, the roll apex wear and the unstable guiding of the single barreled strip becomes more relaxed when splitting a double barreled strip. Szota et al. [24] linked the localized knife apex wear to the operational consistency and the corresponding service life of slitting passes. As a result of stress amplification under the knife collar, the developed wear becomes equivalent to the effective roll’s gap, and hence interrupts the slitting consistency. Localized wear shortens rolling pass workability and operation life. Contrarily, uniform wear of pass groove enhances its durability since it can be compensated easily by reducing rolls’ gap.

## 5. Conclusions

Based on the analysis of results, following conclusions can be outlined:

1. The geometry (W/H) of the strip entering the edging pass is numerically tested by compression using grooved and grooveless dies, and verified by comparing it with the images of produced strips.
2. The FE result of rolling power (185 kW) is found to be in good agreement with the available experimental data (216 kW). This shows the validation of the FE model and boundary conditions.
3. The established FE model is used further to simulate the rolling of a double barreled strip to evaluate its performance in terms of rolling power.
4. The FE results revealed that pre slit rolling of single barrel strip is associated with a power reduction of approximately 12.12%.
5. Higher localized stresses in a preslitting pass at the roll knife of a single barrel strip could cause severe wear of the roll knife apex.

## Figures and Tables

**Table 1:** Chemical composition of the rolled rebar steel (Rolled Egyptian Steel Grade B400B-R).

Element	C	Mn	Si	Cr	Ni	S	P	Fe
Wt. %	0.25	0.55	0.15	0.1	0.1	0.04	0.04	Bal.

## Data Availability

The data presented in this study are available on request from the corresponding author. The data are not publicly available due to the extremely large size.
